# Tonabersat Blocks Gap Junctions and Alleviates Thermal Pain Behavior in Mice

**DOI:** 10.3390/ijms27187987

**Published:** 2026-09-08

**Authors:** Munia Abul Hawa, Rachel Feldman-Goriachnik, Menachem Hanani

**Affiliations:** 1 Laboratory of Experimental Surgery, Hadassah-Hebrew University Medical Center, Mount Scopus, Jerusalem 91240, Israel; 2 Faculty of Medicine, Hebrew University of Jerusalem, Jerusalem 91120, Israel

**Keywords:** gap junctions, liver, tonabersat, pain, trigeminal ganglion

## Abstract

Gap junctions (GJs) are channels that enable exchange of ions and small molecules between cells, and have been implicated in the development and maintenance of neuropathic pain. Injury-induced glial activation in sensory ganglia is associated with increased coupling by GJs, which in turn enhances neuronal excitability, contributing to pain signaling. Tonabersat (TON) was suggested to act as a GJ blocker with analgesic actions, but these claims have been disputed. Here we examined whether TON blocks GJs and whether it influences pain behavior in a mouse pain model. Gap junctional coupling was assayed by the dye coupling method in mouse liver and trigeminal ganglia. Pain behavior was tested in a mouse model of chemotherapy-induced pain, using von Frey filaments (tactile sensitivity), the acetone method (cold sensitivity) and hot plate (heat sensitivity). Intracellular dye injection showed that TON and the GJ blocker carbenoxolone (both 50 µM) inhibited gap junctional coupling by 70% and 82%, respectively. In the trigeminal ganglia, TON and carbenoxolone inhibited gap junctional coupling by 74 and 77%, respectively. TON selectively reduced heat and cold hypersensitivity, but not mechanical threshold. Carbenoxolone reduced all hypersensitivity types. No sex differences were observed. We conclude that both TON and carbenoxolone reduced coupling, indicating their potential to influence GJ-mediated coupling. In behavioral tests TON showed a selective effect for thermal hypersensitivity, but the underlying mechanism is unclear. Carbenoxolone produced a somewhat greater reduction in coupling compared with TON. These findings suggest that GJs play a role in pain pathways and highlight the need to explore whether other pain-relieving drugs act by blocking GJs.

## 1. Introduction

Chronic pain is a serious global health concern with a lifetime prevalence of 12.7–33.7% in the adult European population [1]. Despite an intensive search for therapies, in many cases clinicians do not have adequate tools to treat pain sufferers. The main mode of pain treatment is analgesic drugs, but their efficacy is limited, and they may have some severe side effects. Non-pharmacological pain therapies such as physical exercise and acupuncture have also been explored, but they are not widely used. Most analgesic drugs are directed at neurons, but glial cells have received attention recently as possible candidates as well [2].

Chronic pain usually results from augmented neuronal excitability and also from abnormal intercellular interactions, which are largely mediated by chemical messengers [3]. However, electrical synapses (gap junctions—GJs) between cells are found in the pain pathways, and appear to contribute to pain mechanisms [4,5]. Gap junctions consist of two connexons, which are membrane channels that connect adjacent cells. Each connexon is composed of six units of proteins called connexins (Cxs). There is a large variety of Cxs, and the most abundant types in the nervous system are Cx43, 36 and 32. Most of the GJs in the central nervous system connect glial cells (astrocytes and oligodendrocytes), but some types of neurons are also mutually connected by GJs [6], and neuron–glia coupling has also been observed [7]. In the peripheral nervous system, satellite glial cells (SGCs) [4] and also Schwann cells [8] are mutually coupled by GJs. The expression level and function of GJs in the nervous system can undergo considerable plasticity. For example, in the central nervous system, astrocyte coupling by GJs may increase after nerve damage [5,9,10]. Likewise, in the periphery, various types of injury or inflammation promote SGC–to–SGC, SGC–to-neuron, and neuron–to-neuron coupling by GJ [4,5].

Injury to peripheral nerves is a major cause for chronic pain, and as it can augment GJ-mediated coupling, investigators examined the possibility that GJs may be involved in chronic pain. Indeed, blocking or neutralizing GJs was found to alleviate pain behavior in animal models, confirming this idea [5,11,12,13,14]. Still, to date, there has not been an FDA-approved analgesic drug based on the ability to block GJs, although a clinical trial on a presumed GJ blocker, tonabersat (TON), has been conducted in the context of migraine [15].

Tonabersat is a benzopyran derivative that has been proposed as a drug for treating migraine [16,17]. A possible mechanism underlying TON’s action is blocking GJs [16], but there is no conclusive evidence in the literature that it actually blocks GJs; on the contrary, it was claimed that TON does not block GJs, but does block connexin 43 hemichannels [18]. Thus, the question of the mechanism underlying TON’s action is still open.

In the present work we tested whether TON blocks GJs using the mouse liver as a model because this tissue contains a large number of GJs [19,20]. We also investigated TON’s effect on GJs in the mouse trigeminal ganglia (TG). We carried out a dye coupling study using intracellular injection of a fluorescent tracer, and tested the effect of TON on pain behavior in mice. Our experiments showed that TON blocks GJs and also has analgesic properties.

## 2. Results

### 2.1. Tonabersat Inhibits Gap Junctional Coupling in Mouse Liver

**Figure 1:**
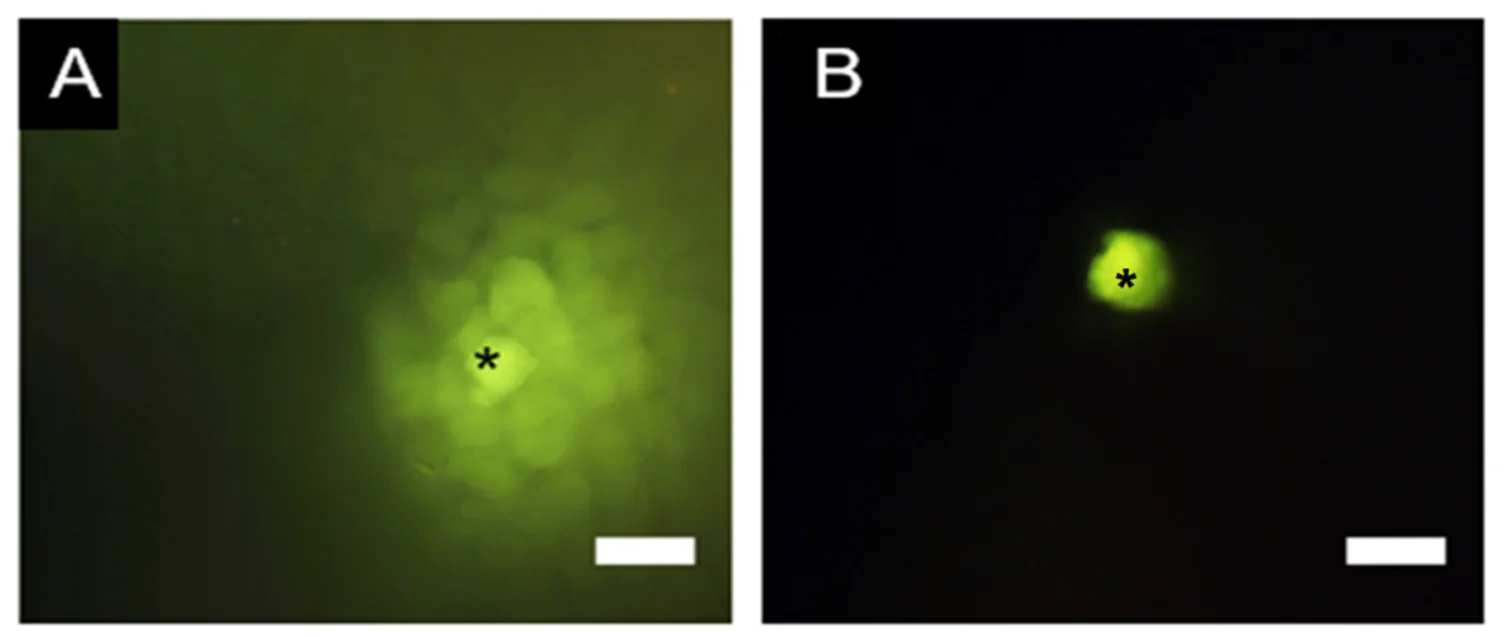
Dye spreads in mouse liver following single cell injection. (**A**) In control, dye injected into a single cell, spreads to multiple neighboring cells, indicating coupling by GJs. (**B**) After TON treatment, the dye was confined to the injected cell. The asterisk marks the injected cell. Scale bar = 30 μm.

**Figure 2:**
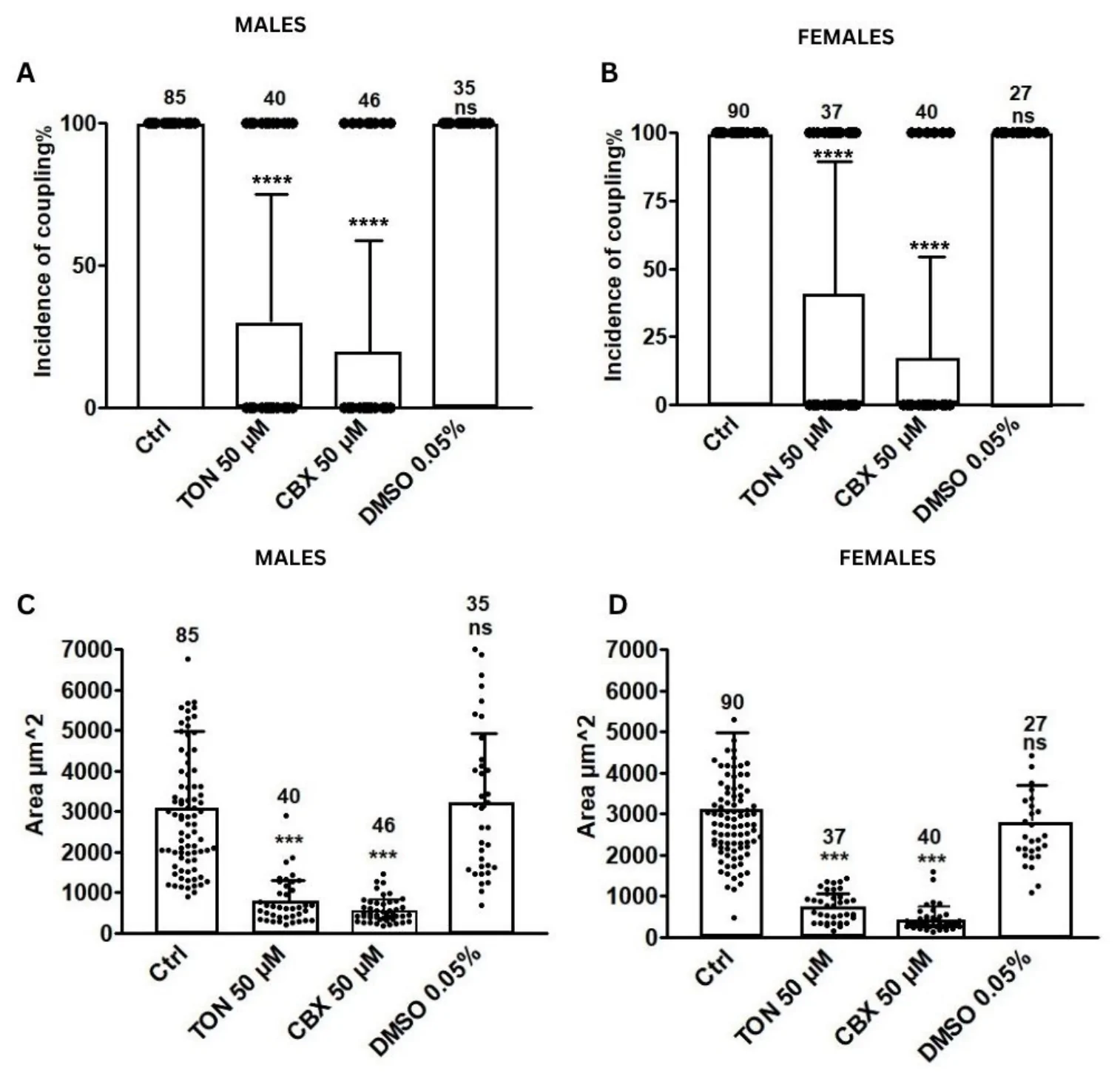
Quantification of tonabersat (TON) and carbenoxolone (CBX) effects on gap junctional coupling in livers of male and female mice. (**A**) Coupling incidence in males. (**B**) Coupling incidence in females. (**C**) Spread area in males. (**D**) Spread area in females. TON and CBX (both 50 μM) significantly reduced coupling compared with control (Krebs) and vehicle (0.05% DMSO), with no sex differences. The number of cells for each experiment is indicated above the bars. Data are presented as mean ± SD. Data were analyzed using one-way non-parametric Kruskal–Wallis test followed by Dunn’s Multiple Comparisons Test. *** *p* < 0.001, **** *p* < 0.0001 vs. control. See text for further details on the calculations. Data for each of the bars were obtained from tissues from four mice; ns, not significant.

The dye injection experiments showed that under control conditions, hepatocytes were dye-coupled extensively (Figure 1). About 20–30 cells were coupled to the injected one, forming a circle with diameter of ~60 µm (Figure 1). Coupling incidence was reduced by 70% after the addition of TON (50 µM) to the bathing medium. In addition to measuring coupling incidence, we calculated the area occupied by the coupled cells, which was also strongly inhibited by TON (Figure 2). Control experiments to test the effect of 0.05% DMSO showed that it had no effect on dye coupling (Figure 2). The results did not differ between male and female mice (Figure 2). The blocking effect of TON on dye coupling was reversed after a wash period of 30–40 min.

For comparison, we tested the effects of the GJ blocker carbenoxolone (CBX, 50 µM), which was also effective in blocking the dye coupling in the liver (Figure 2). To test whether the results are strain-dependent, we repeated the experiments with TON on livers from C57BL/6 mice. The results were similar to those obtained with BALB/c mice, thus validating this work.

### 2.2. Tonabersat Inhibits Gap Junctional Coupling in the Trigeminal Ganglia

**Figure 3:**
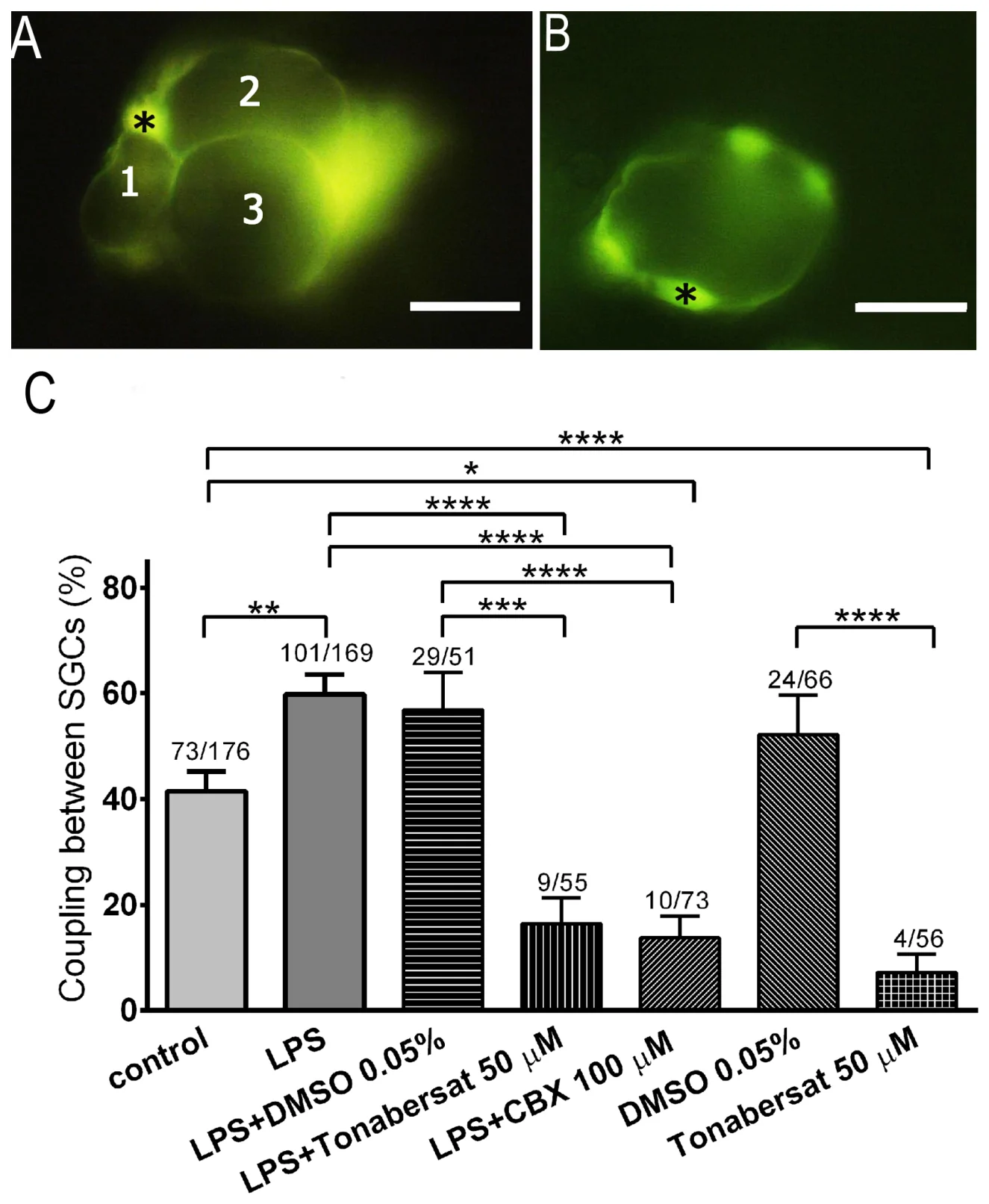
Tonabersat (TON) blocks dye coupling among satellite glial cells (SGCs) in the mouse trigeminal ganglia (TG). (**A**) An example of dye coupling in a TG from a mouse that had been treated a week previously with lipopolysaccharide (LPS). The SGC marked with an asterisk was injected with Lucifer yellow, and the dye passed to SGCs surrounding neurons (1 and 3). (**B**) An example of a result obtained in a TG from an LPS-treated mouse, where the injection was carried out in the presence of TON (50 µM) in the bathing medium. Dye coupling of SGCs is present only around a single neuron. Similar results were obtained with carbenoxolone (CBX, 100 µM). Scale bars, 20 µm. (**C**) Quantitation of the dye coupling data. TON was dissolved in 0.05% DMSO, and the results show that DMSO alone had no effect on the coupling. These data were analyzed using the one-way non-parametric Kruskal–Wallis test followed by Dunn’s Multiple Comparisons Test. * indicates *p* < 0.05; ** *p* < 0.01, *** *p* < 0.001, and **** *p* < 0.0001. The number of ganglia used for each experiment was: control—10, LPS—11, LPS+DMSO—5, LPS+TON—4, LPS+CBX—3, DMSO—4, TON—4. Each ganglion was obtained from a different animal.

The main Cx type in the liver is Cx32 [19,21], and the question arises as to whether TON is a selective Cx32 blocker or acts as a general GJ blocker. To address this question, we carried out a dye coupling study on the mouse trigeminal ganglia (TG), where Cx43 is the prevalent GJ protein [13,14]. We have shown previously that lipopolysaccharide (LPS) induced an increased dye coupling between SGCs in sensory ganglia [12,22], and here we examined whether TON affects this coupling in mouse TG in LPS-treated mice. Figure 3 shows that TON was highly potent in blocking dye coupling between SGCs in these ganglia, indicating that TON is apparently a general GJ blocker.

### 2.3. Tonabersat Reduces Thermal Hypersensitivity

**Figure 4:**
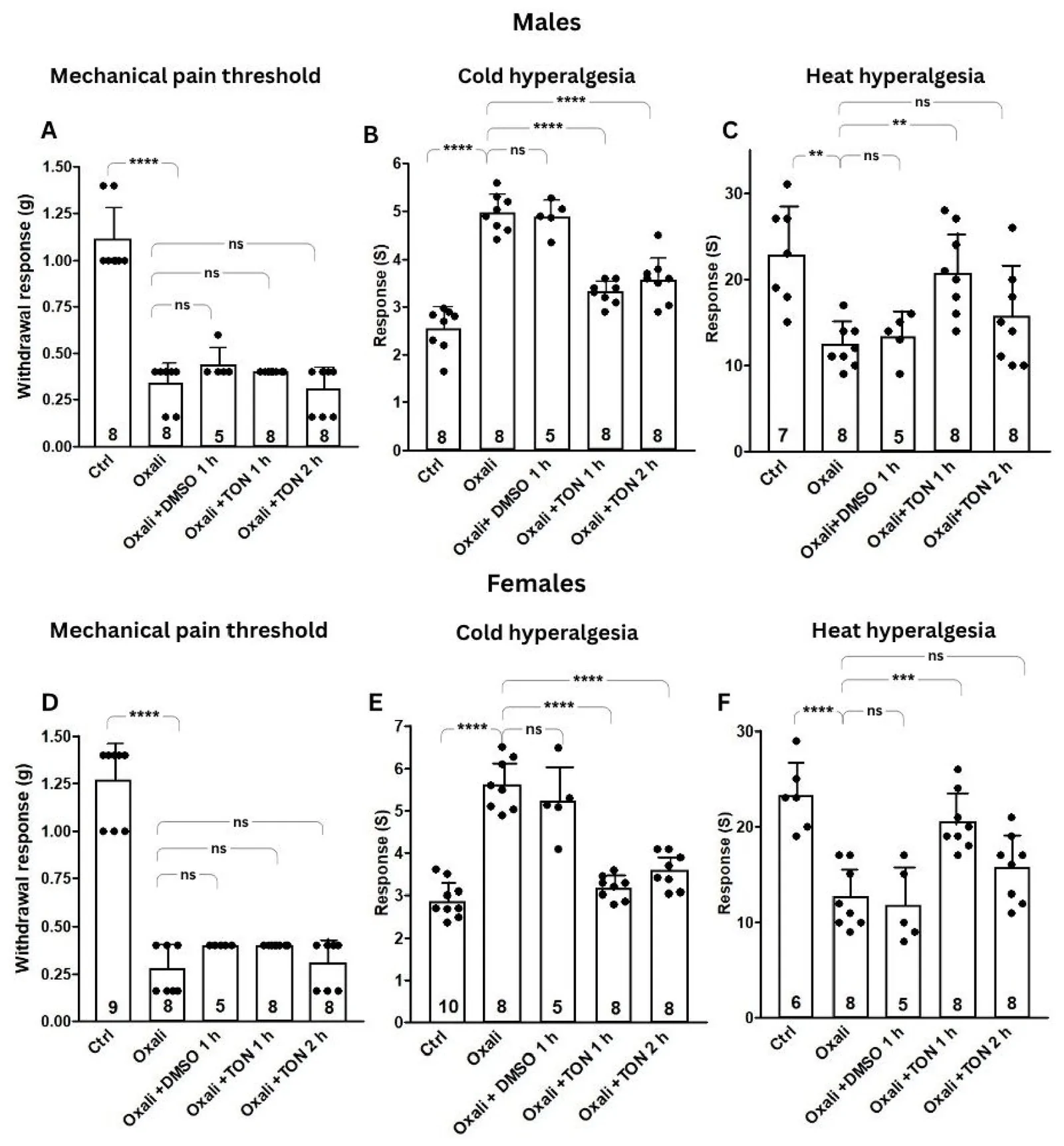
Effects of Tonabersat (50 mg/kg) on pain behavior. Mechanical pain threshold, cold hyperalgesia, and heat hyperalgesia were assessed in male (**A**–**C**) and female (**D**–**F**) mice. In males, TON showed no significant effect on the mechanical pain threshold (**A**), significantly reduced cold sensitivity (**B**), and increased heat withdrawal latency, indicating reduced heat sensitivity (**C**). Similarly, in females, TON had no significant effect on the mechanical pain threshold (**D**), significantly reduced cold sensitivity (**E**), and increased heat withdrawal latency, indicating reduced heat sensitivity (**F**). The number of animals used for each experiment is indicated in the bars. Data are presented as mean ± SD. Statistical analysis was performed using one-way ANOVA, followed by Tukey’s Multiple Comparisons Test. ** *p* < 0.01, *** *p* < 0.001, and **** *p* < 0.0001 compared with the oxaliplatin group; ns, not significant.

We next tested the analgesic properties of i.p. TON by three behavioral assays: tactile sensitivity using von Frey filaments, hot plate, and cold test using the acetone method. As can be seen in Figure 4, oxaliplatin-injected mice displayed greater sensitivity in these three assays compared with untreated mice. TON significantly reduced the behavioral responses in the heat and cold tests in oxaliplatin-treated mice, confirming its analgesic properties. In contrast, in the tactile sensitivity test, TON did not have a significant effect. The results were similar between male and female mice.

**Figure 5:**
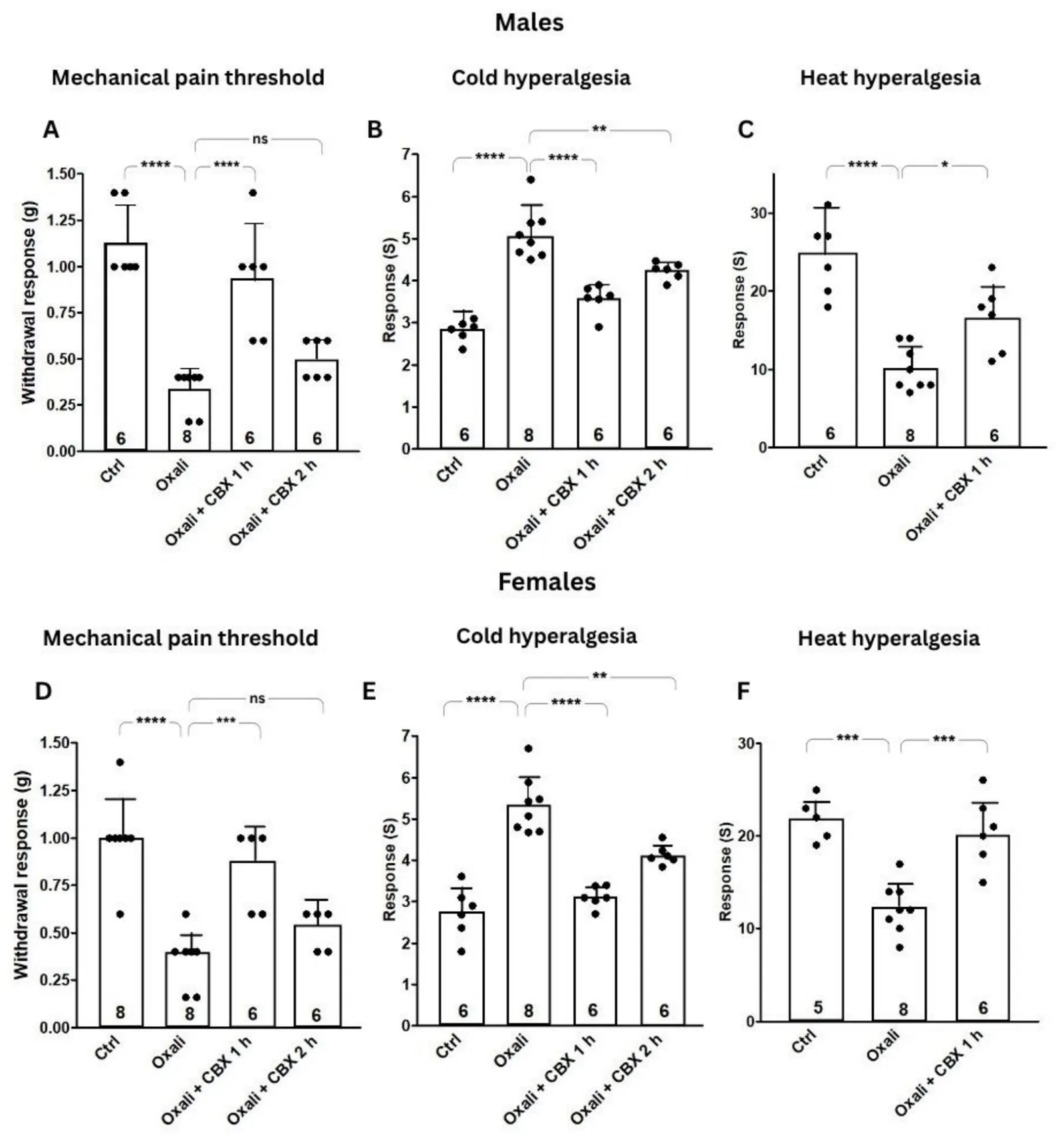
Effects of carbenoxolone (CBX, 50 mg/kg) on pain behavior. Mechanical pain threshold, cold hyperalgesia, and heat hyperalgesia were assessed in male (**A**–**C**) and female (**D**–**F**) mice. In males, CBX significantly increased the mechanical pain threshold (**A**), reduced cold sensitivity (**B**), and increased heat withdrawal latency, indicating reduced heat sensitivity (**C**). Similarly, in females, CBX significantly increased the mechanical pain threshold (**D**), reduced cold sensitivity (**E**), and increased heat withdrawal latency, indicating reduced heat sensitivity (**F**). The number of animals used for each experiment is indicated in the bars. Data are presented as mean ± SD. Statistical analysis was performed using one-way ANOVA, followed by Tukey’s Multiple Comparisons Test. * *p* < 0.05, ** *p* < 0.01, *** *p* < 0.001, and **** *p* < 0.0001 compared with the oxaliplatin group; ns, not significant.

For comparison, we carried out the behavioral tests using i.p. CBX, which has been previously found to reduce tactile sensitivity in mice [11,23]. In all three tests CBX had a clear analgesic action (Figure 5).

## 3. Discussion

The main questions posed in this work were whether TON acts as a GJ blocker and whether it has analgesic actions. Using the dye coupling method in the liver, which is a well-established model for testing GJs, we showed that TON is a potent GJ blocker. We also found that TON alleviates pain behavior in mice, as reported previously [16,17,24].

It was claimed that TON blocks gap junctions [16,17], but there has been no direct experimental evidence to support this. Kim et al. [18] used the scrape loading method to examine whether TON blocks GJs, and found that it did not prevent dye spread, which led to the conclusion that TON is not a GJ blocker. Instead, they suggested that TON blocks Cx43 hemichannels. Obviously, hemichannel blockade would not influence dye coupling, whereas our experiments clearly showed that TON does block them. This disagreement may be explained as follows: One obvious difference between our method and that of Kim et al. [18] is that the scrape loading method is based on damaging cells by breaking their plasma membranes, enabling an extracellular hydrophilic marker to enter the cells, followed by its spreading to neighboring cells by GJs. In contrast, we measured dye coupling by injecting dye from a very fine intracellular micropipette, which causes minimal cellular damage. Secondly, Kim et al. [18] used a human endothelial cell line in culture, whereas we used a fresh intact liver and TG from mice. Thirdly, and most importantly, like us, Kim et al. [18] tested the effect of CBX, and found that it did block the dye spread. However, the concentration of CBX was 200 µM, whereas we used CBX at 50 µM in the liver experiments (100 µM in the TG experiments). Thus, it is quite possible that the cultured endothelial cells possess low sensitivity to GJ blockers, and their coupling was therefore not affected by TON at the concentrations used. These comments do not exclude the possibility that TON blocks hemichannels, but these structures do not connect between cells, and thus do not function as GJs. We conclude that TON is a GJ blocker, as suggested previously.

We noted a differential effect of TON on pain behavior, being effective in alleviating cold and heat hyperalgesia, but not tactile hypersensitivity. We do not have a definitive explanation for this difference, but a survey of the literature revealed that differential drug effects on pain behavior are common and are observed in both humans and experimental animals. For example, Koppert et al. [25] examined the analgesic effect of morphine in humans with skin burn caused by UVB radiation. Subcutaneous injection of morphine alleviated heat, but not mechanical, hyperalgesia in these subjects. The mechanism underlying this phenomenon was not clear. In another study on humans, Weinkauf et al. [26] found in a model of pain induced by nerve growth factor, that lidocaine reduced mechanical hyperalgesia but sensitized responses to heat. These opposing actions were attributed to the blockade of sodium channels in addition to sensitization of TRPV1 channels by lidocaine. A possible way to address this question is that CBX and TON act differentially on ion channels or channels belonging to the transient receptor potential (TRP) superfamily in sensory neurons, such as TRPV1 (heat sensitive) and TRPM8 (cold sensitive). This point may be tested by calcium imaging or by electrical recordings from the neurons. For example, it can be tested whether the responses of TRPV1-positive neurons to capsaicin (TRPV1 agonist) are altered differentially by TON and CBX, or how TRPM8 would respond to cold temperature under the influence of these two drugs.

As can be seen in Figure 4, the analgesic effect of TON lasts less than 2 h. This relative short-lasting effect may be a drawback for clinical use. However, research on humans showed that TON provided relief from migraine for 4 h [17]. Still, this comparison is not straightforward, as TON was given orally to the patients, in contrast to the i.p. administration used in the present work. In any case, the pharmacokinetics of TON needs to be further investigated.

The mechanisms of TON’s actions still need to be explored. Some insight into the question above may be obtained by examining Figure 4 and Figure 5, which show that the effects of TON and CBX on cold pain were mostly similar, whereas for the heat test, TON was more potent than CBX by 19.4% (20/16.75 = 1.194). For mechanical sensitivity, CBX was more potent than TON by 63% (0.9/0.55 = 1.63). In the dye coupling experiment, CBX was clearly more potent than TON. Thus, it is conceivable that inhibition of tactile responses requires a greater GJ blocking potency. This implies that the pathways mediating mechanical pain are less sensitive to GJ blockers, and this idea may be a suitable question for future research.

The main Cx type in the liver is Cx32 [19,21], and it may be asked whether the dye coupling blockade in this tissue is due to a selective action of TON on this specific Cx. The results of Figure 3 indicate that TON also blocks dye coupling among SGCs in the TG, where Cx43 is the dominant Cx [13,14]. Thus, it can be proposed that TON is not a selective GJ blocker, but has a general inhibitory action on GJs, similar to CBX and several other GJ blockers.

There is considerable evidence that in a large number of pain models there is an increased cell coupling by GJs, particularly among SGCs [4], indicating that GJs could serve as suitable targets for pain therapy. For example, CBX reduced visceral pain in mice [11] and diminished acute capsaicin-induced pain in mice [23]. We showed here that two GJ blockers (TON and CBX) have analgesic actions, and previous studies provided similar evidence for other GJ blockers [11,13]. Moreover, other means for blocking GJ such as RNA interference proved to have analgesic effects [14,27]. These observations raise the idea of a possible association between the ability of drugs to block GJs and to display analgesic actions. Testing the analgesic effects of other GJ blockers seems to be a worthwhile idea. Conversely, it would be interesting to examine whether established analgesic drugs act as GJ blockers, which might contribute to their pain-relieving actions.

The present results support the previous claim that TON blocks GJs and also that its analgesic action may be related to GJ blockade. Still, as we injected TON systemically, some caution is required concerning its site(s) of action. Figure 3 shows that TON blocks GJs in mouse TG, in which GJs are upregulated in pain models [4,14], but other targets, including the CNS, are also conceivable and should be examined in future studies. As CBX does not cross the blood–brain barrier, an action on sensory ganglia is highly likely, but TON has a lipophilic nature and may enter the CNS.

In conclusion, we have shown that TON is a general GJ blocker, and can alleviate thermal, but not mechanical, pain. It can be proposed that TON and other GJ blockers may have potential as analgesic drugs.

## 4. Materials and Methods

### 4.1. Animals and Tissue Preparation

Most of the experiments were performed on BALB/c mice (2–5 months old) of either sex; a small number of C57BL/6 mice were also used. The experiments were approved by the Institutional Animal Care and Use Committee and adhere to the guidelines of the International Association for the Study of Pain. Mice were euthanized by CO_2_ inhalation, and livers were rapidly removed and placed into ice-cold, oxygenated Krebs solution containing (in mM): NaCl 118, KCl 4.7, MgSO_4_·7H_2_O 1.2, NaHCO_3_ 14.4, NaH_2_PO_4_·H_2_O 1.2, glucose 11.5, and CaCl_2_ 2.5. Intact liver lobes were pinned onto a chamber with a silicon rubber bottom. The chamber was placed on the stage of an upright Axioskop FS2 microscope (Zeiss, Oberkochen, Germany), equipped with a fluorescence light source and a digital camera linked to a personal computer. During the experiments, the tissue was continuously superfused with oxygenated Krebs solution (95% O_2_/5% CO_2_) at 23–25 °C at flow rate of 1 mL/min to maintain viability and apply drugs. For the experiments on the TG, both ganglia were removed and mounted in a chamber as detailed above.

### 4.2. Intracellular Labeling with Lucifer Yellow

Individual hepatocytes were injected with Lucifer yellow (LY; Sigma, Rehovoth, Israel) 3% in 0.5 M LiCl, from sharp glass microelectrodes (tip resistance 80–120 MΩ) connected to a pre-amplifier (IR 283, Neuro Data Instruments Corp., New York, NY, USA). Dye injection was driven by hyperpolarizing current pulses (100 ms duration, 0.5–1 nA amplitude) delivered at 5 Hz for 1–2 min. Dye coupling was assessed by photographing the cells 1 min after the end of dye injection, and its passage from the injected hepatocyte to neighboring cells was assessed. Dye coupling was quantified by two methods: by the incidence of coupling, and by the area occupied by dye-coupled cells. Dye spread into adjacent hepatocytes indicated functional gap junctional coupling, whereas LY confined to the injected cell denoted uncoupled cells. In the TG experiments, single SGCs were injected with dye in intact ganglia as described previously [22,28]. The GJ blocking effect of TON and CBX was measured from 20 to 120 min after introducing them to the bathing solution. These drugs were present in the solution throughout the experiment. The concentrations that were used were chosen based on our previous work [22].

### 4.3. Behavioral Tests

Oxaliplatin (Tocris, Bristol, UK) was used to induce neuropathic pain; it was prepared at a concentration of 0.9 mg/mL in sterile saline, and mice received two intraperitoneal (i.p.) injections (100 µL each; ~4 mg/kg), spaced three days apart. One week after the first injection, and 1 h before behavioral testing, mice received a single i.p. injection (100 µL) of one of the following: TON (50 mg/kg) dissolved in a 1:3 DMSO/saline solution or CBX (50 mg/kg) dissolved in saline. Control animals received saline, and vehicle control animals received the vehicle. Pain sensitivity was assessed 7 d after the oxaliplatin administration to evaluate the effect of each compound and to confirm the vehicle alone had no analgesic effect. Three types of behavioral tests were performed: responses to mechanical stimulation with von Frey filaments, cold hyperalgesia, and thermal hyperalgesia. Mice were allowed to acclimate for 30 min in the testing area before each procedure.

To evaluate mechanical sensitivity, mice were placed individually in clear plastic chambers positioned on an elevated wire mesh floor. They were allowed at least 20 min to acclimate to the environment before the tests. Mechanical stimulation was applied to the plantar surface of each of the hind paws, using von Frey filaments (Stoelting, Wood Dale, IL, USA) ranging from 0.07 g to 2.0 g, applied in ascending order. Each filament was applied 10 times at intervals of 5 to 20 s, making sure not to stimulate the same spot on the skin repeatedly. A sharp withdrawal of the paw was recorded as a positive response. The withdrawal threshold was defined as the lowest filament force that produced 6 or more positive responses out of 10 trials.

Cold sensitivity was tested using a method adapted from [29]. Mice were placed on a wire mesh and kept under a clear plastic enclosure. A drop of acetone was formed at the tip of a 1 mL syringe (without the needle) and lightly touched against the hind paw’s heel. The acetone quickly spread over the paw surface. The duration of any response, such as paw withdrawal, shaking, licking, or jumping, was noted. If the response lasted less than 1 s, it was recorded as zero. Both left and right hind paws were tested, and the data are presented as the average response time from both sides.

Heat sensitivity was assessed using the hot plate test. Individual mice were placed inside a 400 mL glass beaker, which was placed on the hot plate surface. The plate temperature was maintained at 52 ± 2 °C. The time to a response, such as jumping or paw licking, was recorded as the reaction latency. If the mouse did not respond within 30 s, it was removed from the plate to prevent injury, and a latency of 30 s was recorded.

### 4.4. Chemicals

Tonabersat (Sigma) was dissolved in DMSO, and this solution was diluted with Krebs solution to a final concentration of 50 µM and DMSO 0.05%. Carbenoxolone (CBX, Sigma) was dissolved in distilled water and was diluted to a final concentration of 50 µM. For (i.p.) administration, TON (50 mg/kg) was dissolved in a 1:3 DMSO-to-saline solution, while CBX (50 mg/kg) was dissolved in saline. Control animals received saline, and vehicle control animals received 25% DMSO in 75% saline.

### 4.5. Data Analysis

Fluorescent images were captured at predetermined time points to monitor LY passage between hepatocytes or SGCs. The presence of dye coupling was recorded for each injected cell. The extent of dye spread in the liver was quantified using ImageJ v. 1.53m by measuring the area occupied by the dye-coupled cells. When an LY-injected cell was found to be dye-coupled to other cells, it was marked as 100, and when it was not coupled, as 0. As the data from these experiments had non-normal distribution, we used the one-way non-parametric Kruskal–Wallis test, followed by Dunn’s Multiple Comparisons Test.

Statistical analysis of behavioral data was performed in GraphPad Prism v.6 using one-way ANOVA, followed by Tukey’s Multiple Comparisons Test. Data are presented as the mean ± SD, with significance threshold levels defined as * *p* < 0.05, ** *p* < 0.01, *** *p* < 0.001, and **** *p* < 0.0001.

For the TG experiments, dye coupling data were pooled from multiple experiments. Data were analyzed as for the hepatocytes. When an LY-injected SGC was found to be dye-coupled to SGCs surrounding another neuron, it was marked as 100, and when it was not coupled, as 0.

## Data Availability

The data can be made available by the corresponding authors upon reasonable request.

## Abbreviations

The following abbreviations are used in this manuscript:

CBX	Carbenoxolone
Cx	Connexin
DMSO	Dimethyl sulfoxide
GJ	Gap junction
i.p.	Intraperitoneal
LPS	Lipopolysaccharide
LY	Lucifer yellow
SGC	Satellite glial cell
TG	Trigeminal ganglion
TON	Tonabersat
TRPV1	Transient receptor potential vanilloid 1
